# Efficacy of “Pinggan Formula” in Controlling Acute Type B Aortic Dissection Perioperative Blood Pressure: A Randomized Controlled Clinical Trial

**DOI:** 10.1155/2019/6432953

**Published:** 2019-11-15

**Authors:** Hao Zhang, Danying Zhang, Yudong Sun, Ye Lu, Jiaxuan Feng, Lei Zhang, Chao Song, Zaiping Jing, Chaoqin Yu, Qingsheng Lu

**Affiliations:** ^1^Department of Vascular Surgery, Changhai Hospital, The Navy Military Medical University, Shanghai 200433, China; ^2^Department of General Surgery, No. 202 Hospital of People's Liberation Army, Shenyang 110812, China; ^3^Department of Traditional Chinese Medicine, Changhai Hospital, The Navy Military Medical University, Shanghai 200433, China

## Abstract

**Objective:**

To explore a new treatment that can proceed from the whole, control blood pressure smoothly and coordinate the treatment of multiple factors causing blood pressure fluctuations.

**Method:**

We conducted a single-center, double-blinded, and randomized controlled clinical trial. 48 patients with acute Type B aortic dissection were randomly assigned into two groups: the experimental group, who received pinggan formula treatment, and the control group, who received placebo treatment. The drug was taken orally after meals three times a day. Only when the patients' blood pressure fluctuated, conventional antihypertensive drugs were given to maintain the blood pressure within the target range and the dosage was recorded to convert the DDD value. Meanwhile, the international standardized score was used to evaluate the defecation, sleep, pain, anxiety, and depression of patients in the two groups during the hospitalization.

**Result:**

Univariate analysis was conducted on variables that might affect the assessment results, and it was found that grouping factors had a significant impact on the outcome variables, that is, after the intervention, the mean value of DDDs used in the perioperative period in the control group was 2.19 (0.38, 4.00). (*P*=0.0219), defecation score (2.13 (1.59, 2.67); *P* < 0.0001), sleep score (0.95 (0.40, 1.50); *P*=0.0014), pain score (1.77 (0.61, 2.93); *P*=0.0045), depression score (4.04 (2.95, 5.12); and *P* < 0.0001) were significantly higher than that of the experimental group, and the difference was statistically significant.

**Conclusion:**

Pinggan formula has a clear therapeutic regulation effect on the overall hemodynamics of acute Stanford type B aortic dissection during the perioperative period and can be recommended as an auxiliary drug for conventional antihypertensive drugs at the current stage.

## 1. Introduction

Blood pressure (BP) control is a critical approach to reduce the complication in the perioperative treatment of acute Type B aortic dissection [1]. The rise of blood pressure or its fluctuation is related to multiple factors, including pain, constipation, anxiety, and insomnia. The majority of previous therapies are pure symptomatic treatment, which often requires a combined use of multiple drugs and 24-hour BP monitoring. Each of the traditional antihypertensive drugs has its own effective pathway and single action treatment, and long-term combined use is easy to cause negative interactive effects and blood pressure fluctuation, especially for uncontrollable recurrent hypertension, it is difficult to maintain stable blood pressure. Poor blood pressure control increases perioperative mortality in acute type B aortic dissection [2]. At present, endovascular therapy is the main surgical method for the treatment of such diseases, and it has advantages of minimal invasiveness and quick recovery rate; however, studies have shown that blood pressure fluctuation is an important risk factor of a postoperative adverse event [3]. From a unique herbal medicine perspective, Chinese herbal treatment discovers that this type of patients belongs to “ascendant hyperactivity of liver yang” symptom. “Pinggan formula” is an herbal prescription which is invented specifically to treat perioperative symptoms and is directed at pathogenesis of aortic dissection. The treatment principles of liver-pacifying and wind-extinguishing, heat-clearing and blood activation to regulate the bowels, and liver-tonifying and kidney-replenishing can together act on constipation, pain, insomnia, and other factors and help to control blood pressure.

In this research paper, we examine the following hypothesis, namely, the application of “Pinggan formula” assisted blood pressure control therapy can (1) reduce the defined daily dose (DDD) of common antihypertensive drugs, (2) mediate factors such as constipation and pain and synergistically control the blood pressure, and (3) meet the blood control standards of Stanford type B aortic dissection under the condition of reduced amount of common antihypertensive drugs.

## 2. Methods

### 2.1. Design of Experiment

This experiment is a parallel, random, double-blind controlled study conducted at Shanghai Changhai Hospital (experiment and control groups were assigned randomly in a 2 : 1 ratio). All patients in this study gave their written informed consent.

### 2.2. Object of Study

This study started on January 2016 at Shanghai Changhai Hospital vascular surgery department, and all subjects were administrated to in-hospital treatment by outpatient and emergency department.

The inclusion criteria are as follows: (1) the diagnosis met the criteria of acute Stanford type B aortic dissection and (2) initial onset.

The exclusion criteria are (1) atypical aortic dissection, including aortic wall hematoma, and aortic ulcer, (2) after aortic surgery, (3) branch artery ischemia or near rupture (pleural effusion, hemoptysis, or radiographically encapsulated rupture), (4) retrograde tear involving ascending aorta, (5) pregnancy, (6) obvious liver impairment (AST/ALT > 200 mg/dl, total bilirubin > 40 mg/dl, and albumin < 30 mg/dl), and (7) obvious renal impairment (creatinine > 200 mmol/l and urea nitrogen > 50 mg/dl).

### 2.3. Calculation of Sample Size

According to the results of the preliminary experiment, the main observational indicators (the mean value of DDDs) were 10.1 ± 6.8 and 18.6 ± 4.6 in the experimental and control group, respectively. The experimental group and control group were matched in a 2 : 1 ratio, and a two-sided test was adopted, when *α* = 0.05; 84% effectiveness could be reached if there were 6 cases from the control group and 12 cases from the experimental group. Considering the possibility of dropping out and increasing the power, the sample size of the trial is expanded to 16 cases in the control group and 32 cases in the experimental group. A total of 48 patients need to be enrolled in this study, which is expected to be completed within 1 year.

### 2.4. Random Grouping

While recruiting subjects, independent third-party researchers used statistical software SPSS 18.0 and generated random number codes in a 2 : 1 ratio between the experimental and the control group. Both “Pinggan formula” and placebo were in granule form with the same appearance and taste. They were packaged in bags and numbered consecutively. Patients were randomly assigned by the envelope method and received a preprepared drug with the corresponding number.

### 2.5. Blind Method

The subjects and researchers, including statisticians, outcome assessors, and data analysts, were unaware of the grouping information. Although the third party of the experiment was aware of the grouping information, they did not participate in the result evaluation or data analysis and were only responsible for the management of the distribution and recycle of the experimental drugs and the establishment of detailed records of drug distribution. In addition, all the researchers were trained on detailed procedures before the trial began, and they were asked to adhere strictly to the separation principle.

### 2.6. Intervention Measures

Interventions started after subjects were enrolled and sequentially numbered. All patients were given Chinese herbal preparations according to their own number; the drug was taken orally after meals three times a day. Only when the patients' blood pressure fluctuated, that is, continuously (more than 15 minutes) exceeded the upper limit of blood pressure values (systolic and diastolic blood pressure were 120 mmHg and 80 mmHg, respectively), conventional antihypertensive drugs were given to maintain the blood pressure within the target range (systolic and diastolic blood pressure were 90–120 mmHg and 60–80 mmHg, respectively) and the dosage was recorded to calculate the DDD value. These hemodynamic parameters were monitored in a real-time manner by the ward EGC monitor.

#### 2.6.1. Experiment Group

Each dose of “Pinggan formula” was decocted with a combination of Chinese herbs (9 g Rhizome Gastrodiae, 18 g Ramulus Uncariae Cum Uncis, 15 g Concha Haliotidis, 9 g Fructus Gardenial, 9 g Radix Scntellanae, 15 g Eucommiae, 15 g Radix Cyathulae, 15 g Radix Achyranthis Bidentatal, 15 g Tuber Fleeceflower Stem, 15 g Semen Ziziphi Spinosae, 9 g Pericarpium Citri Tangerinae, 15 g Poria, 15 g Radix Rubiae, 6 g Radix et Rhizomea, 9 g bamboo leaves, 9 g frankincense, and 9 g myrrh), and granules were then prepared and given three times a day after meals.

#### 2.6.2. Control Group

The placebo of the control group was provided by the research center. The placebo in the control group was made of food coloring, maltodextrin, and lactose, with the same taste and dose as “Pinggan formula” and almost had no pharmacological effects. The administration method was also taking it three times a day after meals and making it feel the same as the experimental group.

The dosage of granules in the experimental group and the placebo in the control group were both 27.8 g after the drug ingredients were concentrated.

### 2.7. Quality Control

In the course of this study, a third-party inspector designated by the sponsor has been conducting regular on-site inspection visits to ensure that all contents of the study program were strictly observed and the research data were filled in correctly. Research participants were trained to unify recording methods and judgment criteria. The entire clinical trial process should be conducted under strict procedures. All observations and findings in clinical trials should be verified to ensure the reliability of data, to ensure that all conclusions were derived from the original data, and that appropriate data management measures were in place during clinical trial and data processing stages.

### 2.8. Evaluation Method

Baseline conditions such as age, BMI, and gender were analyzed for the enrolled subjects. The dosage and administration time of antihypertensive agents for the two groups were carefully recorded during the patients' in-hospital stay. According to the defined daily dose (DDD) of antihypertensive drugs set by the WHO, the antihypertensive drug use frequency (DDDs) was calculated as daily consumption/DDD, the cumulative daily consumption of conventional antihypertensive drugs was obtained by summing the daily antihypertensive drug use frequency [4]. For antihypertensive drugs with no established DDD value, the daily dose standard in the drug specification was used to establish the DDD value. Meanwhile, the international standardized score was used to evaluate the defecation, sleep, pain, anxiety, and depression of patients using PAC-SYM (patient assessment of constipation symptom), PSQI (Pittsburgh sleep quality index), VAS (visual analogue score), SAS (self-rating anxiety scale), and SDS (self-rating depression scale), in the two groups during the hospitalization.

### 2.9. Statistical Analysis

Basic data of the two groups of patients (age, BMI, length of hospital stay, cardiac ejection fraction, gender, use of antihypertensive drugs, previous medical history, etc.) were analyzed by variance to ensure that the baseline data of the two groups of randomly assigned patients were consistent. The scores of routine antihypertensive drug dosage and defecation, sleep, pain, anxiety, and depression during hospitalization were recorded. Intervention factors, age, gender, BMI, and hypertension history were taken as independent variables to establish a univariate analysis model, and the changes of the above indicators were observed. In order to avoid the statistical error caused by confounding variables, multivariate regression analysis was conducted after adjusting and controlling the variables such as age, gender (male, female), BMI, whether avulsion was reversed (no, yes), and history of hypertension (no, yes) in the two groups to confirm the results of univariate analysis. All statistical analyses were performed using EmpowerStats software.

## 3. Results

### 3.1. Number of Participants

**Figure 1:**
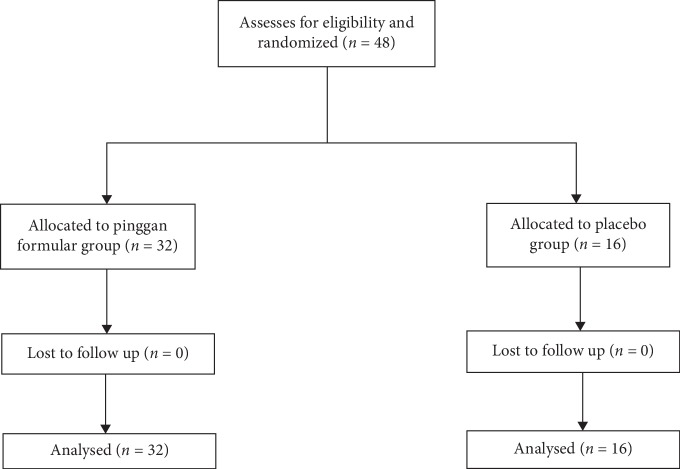
Participant flowchart.

A total of 48 patients with acute type B aortic dissection (42 males and 6 females) were included in the study from January to December 2016 and were divided into experimental and control groups in a 2 : 1 ratio (Figure 1). Among the enrolled patients, 1 case (3.1%) of dissection rupture occurred in the experimental group and died. In the control group, 1 patient (6.2%) underwent emergency surgery and was cured. The condition of 2 cases (6.2%) in the experimental group and 1 case (6.2%) in the control group turned stable and cured after symptomatic treatment, and the patients were discharged without surgical treatment. In the experimental group, 3 patients (9.4%) had severe diarrhea due to the adverse effect of drugs, and the dosage was reduced with the approval of the project leader. Before drug dosage reduction and discontinuation, the observational indicators of patients in the two groups during hospitalization could still serve as effective indicators, so the number of patients eventually included in the study remained unchanged (Figure 1).

### 3.2. Baseline Characteristics

The patients' baseline characteristics are shown in Table 1. The mean age of enrolled patients was 57.6 years (36–85 years), and the mean length of hospital stay was 11.5 days (3–24 days). There were no significant differences between the two groups in age, BMI, length of hospital stay, cardiac ejection fraction, gender, use of antihypertensive drugs (whether or not and the number of drugs used), and chronic medical history (there were no significant differences between the groups in demographics or patient history).

### 3.3. Clinical Outcomes

After “Pinggan formula” and placebo intervention, the use of routine antihypertensive drugs (DDDs) during perioperative hospitalization differed between the two groups, the DDD value of the control group was higher than that of the experimental group (*β*=2.19) with statistical significance (*P*=0.0219), and the monitor showed that the blood pressure was maintained within the control range (systolic and diastolic blood pressure were 90–120 mmHg and 60–80 mmHg, respectively). This result indicated that “Pinggan formula” can effectively reduce the dosage of routine antihypertensive drugs in the absence of obvious fluctuation of patients' blood pressure. The results of the observational indicators showed that, when conventional antihypertensive drugs were used, the intervention of “Pinggan formula” and placebo produced significant differences in the evaluation results of some factors that might cause blood pressure fluctuations between the two groups (sleep: 0.95, *P*=0.0014; pain: 1.77, *P*=0.0045), that is, the control and regulation of insomnia and pain that may cause fluctuations in blood pressure in the experimental group were significantly better than that in the control group. At the same time, it was found that age, gender, BMI, history of hypertension, etc., did not cause significant differences between the two groups in terms of evaluation indicators (antihypertensive drug dosage, defecation, pain, depression, anxiety, and sleep).

### 3.4. Statistical Analysis

Compared with experimental group, there was no statistical difference in the DDDs value, sleep index, pain index, and anxiety index at the initial baseline prior to the intervention of the experimental drug “Pinggan formula” and placebo, and there were statistical differences in defecation index (4.7 ± 2.5 vs. 6.2 ± 1.6; *P*=0.015) and depression index (26.8 ± 2.3 vs. 30.1 ± 1.7; *P* < 0.001) at the initial baseline (Table 2).

Univariate analysis was conducted on variables that might affect the assessment results, and it was found that grouping factors had a significant impact on the outcome variables, that is, after the intervention, the mean value of DDDs (2.19 (0.38, 4.00); *P*=0.0219), defecation score (2.13 (1.59, 2.67); *P* < 0.0001), sleep score (0.95 (0.40, 1.50), *P*=0.0014), pain score (1.77 (0.61, 2.93); *P*=0.0045), and depression score (4.04 (2.95, 5.12); *P* < 0.0001) were significantly higher than that in the experimental group, and the difference was statistically significant. But there were statistical differences in defecation and depression at the initial baseline (Table 2), so the outcomes after the intervention cannot explain the significant impact of grouping factors. Anxiety score of the control group (−0.65 (−1.50, 0.21); *P*=0.1438) was lower than that of the experimental group, but the difference was not statistically significant (Table 3).

The mean DDDs, defecation score, sleep score, pain score, anxiety score, and depression score between the two groups were not significantly affected by age, gender, BMI, dissection avulsion, hypertension, or other factors (Table 3).

In order to exclude the error and influence of confounding variables on statistical results, all patients underwent multiple regression analysis after controlling variables such as age, gender, BMI, retrogradation level, and history of hypertension, adjusted model was obtained as shown in Table 4, different intervention factors caused significant differences in DDDs value, defecation, sleep, pain, and depression in the two groups, i.e., under the control of patients' blood pressure, the dose of antihypertensive drugs in the experimental group was significantly lower than that in the control group, and the control of constipation, insomnia, pain, depression, and other factors that may affect the fluctuation of blood pressure was also better than that in the control group [5–8]. This result was consistent with the unadjusted result, indicating that other variables have no significant influence on the evaluation results other than the intervention factors of experimental grouping (Table 4).

## 4. Discussion

The main purpose of this study is to observe the control effect of traditional Chinese medicine “Pinggan formula” on perioperative blood pressure fluctuation of acute Stanford type B aortic dissection and its effect on assisting treatment of related risk factors that may cause blood pressure fluctuation.

Acute Stanford type B aortic dissection mainly relies on surgical treatment and requires a long perioperative period. Uncontrollable hypertension, hypotensive, blood pressure fluctuation, pain, and other factors during the perioperative period can cause hemodynamic abnormalities and increase the incidence of adverse events such as vascular rupture and death [9]. Based on the above facts, it is crucial to strictly control the patient's blood pressure within the safety range during the perioperative period, and at the same time, it is necessary to closely monitor the blood pressure to avoid fluctuations. At present, the main measure to control blood pressure is taking a variety of conventional antihypertensive drugs, in combination with other symptomatic drugs for treatment, as well as the 24-hour strict blood pressure monitoring. Although antihypertensive drugs can control blood pressure within a certain range, due to the specific side effects of drug's own mechanism of action and different drug delivery time intervals, fluctuations in patients' blood pressure is inevitable. In addition, patients often take such drugs for a long time before hospitalization and thus form drug resistance. During hospitalization, the dosage of vasoactive drugs is relatively large and the drug effect is weakened, so new drugs need to be given to assist in controlling blood pressure. Moreover, the majority of patients are accompanied by pain, insomnia, anxiety, depression, constipation, and other symptoms that may cause fluctuations in blood pressure [10, 11]. For these risk factors, only relevant drugs can be used for symptomatic treatment. However, different administration time of these drugs, their different effects, and lack of overall planning will aggravate the sudden rise and fall of blood pressure and seriously threaten the perioperative safety of patients. Clinicians are in urgent need of new regulatory treatment methods that can proceed from the whole, control blood pressure smoothly, and coordinate the treatment of multiple factors causing blood pressure fluctuations.

Under the guidance of the theory of Yin–Yang and five elements, Traditional Chinese Medicine (TCM) studies physiology, pathology, and pharmacology of the human body and their relationship with the natural environment from a dynamic and holistic perspective, seeking the most effective way to prevent and cure diseases [12]. According to the theory of TCM, aortic dissection is located in the vein and the disease is a mixture of deficiency and solid; solid is mostly related to phlegm, blood stasis, dampness and heat, and deficiency is mostly due to the deficiency of Qi, blood, and Yin and Yang. Clinical studies have found that patients with acute Stanford type B aortic dissection will have severe chest pain, headache, dizziness, restlessness, insomnia, dreaminess, constipation, and other symptoms during the perioperative period, as well as the appearance of dark tongue, slightly yellow coating, and pulse string number. These syndromes can be summarized as a differentiation in traditional Chinese Medicine theory: the disease occurred in middle energizer, and was due to deficiency of the liver and kidney, ascendant hyperactivity of liver yang, extreme heat engendering wind, and stasis of phlegm. Ascendant hyperactivity of liver yang and harassing of wind yang caused headache and dizziness, residual of liver yang, heat harassing caused heart spirit harassing, insomnia, and dreaminess. These signs are in line with the characteristics of traditional Chinese medicine of aortic dissection, and in view of the above symptoms and representations, the treatment principle of “calming the liver to extinguish wind, clearing heat and activating blood to clear the bowels, and tonifying the liver and kidney” should be adopted.

There are many herbal ingredients in “Pinggan formula,” including: *Gastrodia elata*, *Uncaria*, concha haliotidis, *Rheum officinale*, radix cyathulae, madder, frankincense, myrrh, *Eucommia ulmoides Oliv*, *Lophatherum gracile*, *Gardenia*, *Scutellaria*, caulis polygoni multiflori, and wild jujube seed. The “Pinggan formula” can clear the liver heat and liver yang, extinguish wind to arrest convulsions, pacify the liver to subdue yang, remove accumulation with purgation, clear the heat and purge fire, detoxicate and cool the blood, unblock the meridian and dissipate stasis, conduct blood downward, induce diuresis, cool and activate blood, dispel stasis, unblock the meridian, move Qi and relieve pain, tonify and replenish the liver and kidney, fortify the spleen and invigorate the stomach, and nourish the heart to tranquilize through mixing the ingredients in their proper proportion.

In clinical practice, on the basis of effectively maintaining stable blood pressure, “Pinggan formula” can also improve constipation, insomnia, pain, depression, and other risk factors that may cause fluctuations in blood pressure, so as to effectively regulate the perioperative blood pressure of acute aortic dissection as a whole. “Pinggan formula” has a clear therapeutic effect on constipation, which may be the cause of severe, intolerable diarrhea in 3 patients in the experimental group. The constipation of the remaining patients was improved without severe diarrhea.

In the experiment of perioperative blood pressure control of acute type B aortic dissection with the assistance of Chinese medicine “Pinggan formula,” the comfort and living quality of the patients in the experimental group during the hospitalization were significantly improved, symptoms associated with aortic dissection such as insomnia, pain, constipation, and anxiety were significantly reduced, and the intensity of routine antihypertensive drugs (DDDs value) was significantly decreased. It can be considered that “Pinggan formula” has an advantage in synergistic control of perioperative blood pressure in patients with acute type B aortic dissection.The drug used in this experiment is a granular preparation made according to the Chinese characteristics. In the future, we aim to study Chinese medicine preparations suitable for Western human characteristics to regulate the blood pressure smoothly and safely.

## 5. Conclusion

The study shows that the traditional Chinese medicine “Pinggan formula” can significantly reduce the dose of conventional antihypertensive drugs (DDDs value) while maintaining stable perioperative blood pressure (systolic blood pressure and diastolic blood pressure at 90–120 mmHg and 60–80 mmHg, respectively) in patients with acute Stanford type B aortic dissection. At the same time, it can regulate and control pain, constipation, insomnia, and other factors that may cause blood pressure fluctuations and improve corresponding symptoms. That is to say, Pinggan formula might be an ideal Chinese medicine preparation for blood pressure control during the perioperative period of aortic dissection and can be recommended as an auxiliary drug for conventional antihypertensive drugs at the current stage.

## Figures and Tables

**Table 1:** Baseline characteristics of patients with type B acute aortic dissection.

Characteristics	Experimental group	Control group	*P* value
*N*	32	16	
Age (year)	58.16 ± 12.62	56.44 ± 12.06	0.654
BMI (kg/m^2^)	26.06 ± 4.82	24.93 ± 3.02	0.398
Hospital stays (day)	10.88 ± 4.02	12.69 ± 4.99	0.181
Ejection fraction (%)	62 ± 4	62 ± 3	0.779
Female sex no. (%)	4 (12.50)	2 (12.50)	1.000
Hypertension no. (%)	23 (71.88)	12 (75.00)	0.818
Diabetes no. (%)	2 (6.25)	1 (6.25)	1.000
Hyperlipidemia no. (%)	3 (9.38)	1 (6.25)	0.712
Peripheral vascular disease no. (%)	0 (0.00)	1 (6.25)	0.153
Stroke no. (%)	1 (3.12)	2 (12.50)	0.206
Coronary heart disease no. (%)	3 (9.38)	0 (0.00)	0.206
Antihypertensive drugs no. (%)	13 (40.62)	7 (43.75)	0.836
Number of antihypertensive drugs no. (%)			0.190
0	19 (59.38)	9 (56.25)	
1	7 (21.88)	7 (43.75)	
2	3 (9.38)	0 (0.00)	
3	3 (9.38)	0 (0.00)	
Aortectasia no. (%)	3 (9.38)	1 (6.25)	0.712
Renal insufficiency no. (%)	1 (3.12)	3 (18.75)	0.065

**Table 2:** Baseline characteristics of evaluation index.

Evaluation index	Experimental group	Control group	*P* value
DDDs	10.26 ± 10.45	8.87 ± 5.80	0.558
Defecation score	4.70 ± 2.50	6.20 ± 1.60	0.015
Sleep score	5.90 ± 1.40	7.00 ± 1.90	0.054
Pain score	6.50 ± 3.00	7.40 ± 2.50	0.309
Anxiety score	29.60 ± 1.30	29.00 ± 2.10	0.306
Depression score	26.80 ± 2.30	30.10 ± 1.70	<0.001

**Table 3:** Results of single factor analysis on evaluation index.

	DDDs		Defecation score		Sleep score		Pain score		Anxiety score		Depression score	
	*β* (95% CI)	*P* value	*β* (95% CI)	*P* value	*β* (95% CI)	*P* value	*β* (95% CI)	*P* value	*β* (95% CI)	*P* value	*β* (95% CI)	*P* value
Group	2.19 (0.38, 4.00)	0.022	2.13 (1.59, 2.67)	<0.001	0.95 (0.40, 1.50)	0.001	1.77 (0.61, 2.93)	0.005	−0.65 (−1.50, 0.21)	0.144	4.04 (2.95, 5.12)	<0.001
Age	−0.04 (−0.11, 0.04)	0.322	0.01 (−0.02, 0.04)	0.648	−0.01 (−0.03, 0.02)	0.560	0.03 (−0.01, 0.08)	0.180	0.01 (−0.02, 0.04)	0.533	−0.02 (−0.08, 0.04)	0.520
Female sex	−0.88 (−3.60, 1.85)	0.531	0.45 (−0.71, 1.61)	0.447	−0.35 (−1.22, 0.52)	0.430	0.06 (−1.75, 1.87)	0.945	−0.98 (−2.20, 0.23)	0.120	−0.56 (−2.82, 1.71)	0.631
BMI	0.08 (−0.13, 0.29)	0.474	−0.07 (−0.16, 0.01)	0.104	0.04 (−0.03, 0.11)	0.254	−0.09 (−0.23, 0.05)	0.207	−0.02 (−0.12, 0.07)	0.626	−0.00 (−0.18, 0.18)	0.989
Hypertension	−0.47 (−2.50, 1.56)	0.653	−0.04 (−0.91, 0.82)	0.921	0.19 (−0.46, 0.83)	0.578	0.48 (−0.86, 1.82)	0.484	−0.60 (−1.51, 0.31)	0.203	0.60 (−1.08, 2.28)	0.486

**Table 4:** Results of multivariate regression analysis on evaluation index.

	Nonadjusted		Adjust	
	*β* (95% CI)	*P* value	*β* (95% CI)	*P* value
*Y* = DDDs				
Group	2.19 (0.38, 4.00)	0.022	2.74 (0.72, 4.76)	0.011
*Y* = defecation score				
Group	2.13 (1.59, 2.67)	<0.001	1.99 (1.47, 2.52)	<0.001
*Y* = sleep score				
Group	0.95 (0.40, 1.50)	0.001	1.02 (0.39, 1.64)	0.003
*Y* = pain score				
Group	1.77 (0.61, 2.93)	0.005	1.56 (0.28, 2.84)	0.022
*Y* = anxiety score				
Group	−0.65 (−1.50, 0.21)	0.144	−0.49 (−1.35, 0.36)	0.262
*Y* = depression score				
Group	4.04 (2.95, 5.12)	<0.001	4.21 (3.01, 5.41)	<0.001

Adjust: adjust for age; gender (male, female); BMI; retrograde aortic dissection (no, yes); hypertension (no, yes).

## Data Availability

The data used to support the findings of this study are available from the corresponding author upon request.
